# Response to “A reappraisal of the phylogenetic placement of the *Aquilegia* whole-genome duplication”

**DOI:** 10.1186/s13059-020-02211-z

**Published:** 2020-12-08

**Authors:** Gökçe Aköz, Magnus Nordborg

**Affiliations:** 1 Vienna Graduate School of Population Genetics, Vienna, Austria; 2 Gregor Mendel Institute, Austrian Academy of Sciences, Vienna Biocenter, Vienna, Austria

We were asked to respond to the correspondence of Tao Shi and Jinming Chen and agreed to do so because we feel open and frank communication is good for science. We note from the outset that neither we nor anyone else knows what happened over 100 million years ago and that we make no “assertions” in our original article, merely analyze and interpret the data produced by evolutionary history to the best of our abilities. Our understanding of ancient events improves with the availability of data and improved analysis methods, and our article was written in this spirit. In particular, we argued that conserved genome organization (synteny) better preserves information about ancient events than traditional methods based on gene-by-gene comparisons. At the same time, we agreed with one reviewer that our analysis methods were ad hoc and that there is a need for a more rigorous framework. As data of this type continue to accumulate, we are hopeful that appropriate models will be developed.

The correspondence by Shi and Chen has two parts. First, they argue that our results are due to homoplasy—repeated, independent occurrences of the same change leading to independent branches of a phylogenetic tree being put together. As they correctly note, it is well known that rate heterogeneity in sequence evolution can cause problems in phylogeny through this mechanism. However, they do not explain how heterogeneity in gene loss could cause the pattern we observe, in which there is a pairwise correspondence between duplicated segments between species (our Fig. 5). This would seem to be exceedingly unlikely to happen by chance (although we reiterate that more modeling in this area is needed). Shi and Chen also seem to suggest that parallel selection could lead to convergent evolution. This is true by definition, but we again fail to see how such selection would operate. Similarly, they argue that a chromosomal fusion shared between *Aquilegia* and *Vitis* (and *Theobroma*, although they do not mention this) is more likely due to independent events than shared ancestry. This is hard to evaluate given the information provided and is in any case not central to our conclusion.

Second, Shi and Chen use gene-based analyses to support independent polyploidization in *Aquilegia*. Based on the distribution of pairwise sequence divergence between gene duplicates (Ks), they argue that copies within *Aquilegia* are more similar to each other than they are to copies in other species, suggesting that duplication happened within *Aquilegia.* However, this difference seems largely to be due to a tail of extremely closely related copies in *Aquilegia* (their Fig. 2c,d), which probably do not reflect ancient events. More generally, as noted in our paper, we think comparing pairwise sequence divergence between copies separated by over 100 million years of evolution is fraught with error and mostly uninformative. The same concern applies to their second analysis, which uses traditional phylogenetic tree-building across 700 individual genes to argue that independent duplication is more likely.

Time will tell who is right. We are optimistic that the increasing availability of complete genomes will lead to better understanding of both phylogeny and genome evolution.

